# Disparities in time to breast cancer surgery in New Zealand by level of neighbourhood deprivation: a population-based study

**DOI:** 10.1007/s10552-025-02032-0

**Published:** 2025-07-22

**Authors:** Leah Boyle, Olivia M. Parker, Sandar Tin Tin

**Affiliations:** 1 Department of General Surgery, Hutt Hospital, Wellington, New Zealand; 2 Cancer Epidemiology Unit, Nuffield Department of Population Health, University of Oxford, Oxford, UK; 3 Wellington Hospital Intensive Care Unit, Wellington, New Zealand; 4 Epidemiology and Biostatistics, School of Population Health, University of Auckland, Auckland, New Zealand

**Keywords:** Deprivation, Breast cancer, Inequities, Delay, Surgery

## Abstract

**Purpose:**

The New Zealand (NZ) Faster Cancer Treatment (FCT) plan aims for equitable cancer treatment irrespective of sociodemographic factors. Research on its impact on breast cancer surgery times is limited. This study evaluates whether (1) there are differences by level of neighbourhood deprivation in time to surgery in women with early-stage (1–3a) breast cancer in NZ between 2000 and 2020 and (2) whether this association differs pre- and post- FCT implementation.

**Methods:**

This retrospective analysis used *Te Rēhita Mate Ūtaetae* (NZ Breast Cancer Foundation National Register), a prospectively maintained national database of breast cancers. Logistic regression models evaluated differences by neighbourhood deprivation in time to surgery beyond 31 days (defined in the FCT as the longest acceptable delay in time to first treatment). Deprivation was measured using the NZ Deprivation (NZDep) Index, an area-based measure of socioeconomic deprivation in deciles (decile 1 = least deprived to decile 10 = most deprived) categorised into quintiles. Models were adjusted sequentially for potential contributing factors across five domains; demographic [age, ethnicity, urban or rural place of residence], mode of diagnosis [screening programme or symptomatic], tumour [stage, grade, receptors], treatment facility type [public/private hospital] and treatment [locoregional and systemic]. Subgroup analysis by pre- and post-FCT implementation date were undertaken.

**Results:**

Of the 20,322 women included in the analysis, 23.5% were in the least deprived neighborhoods (NZDep index 1–2) and 13.8% were in the most deprived neighborhoods (NZDep index 9–10) and 22.3% 21.0% 19.5% were in 3–4, 5–6 and 7–8, respectively. Overall, 73% of the women were NZ European, 10% Māori (indigenous NZ people), 7% Pacific (from the Pacific islands) and 10% were Asian. In the unadjusted model, compared to the least deprived quintile, all other NZDep index quintiles were more likely to experience delay beyond 31 days. In the maximally adjusted model, compared to the least deprived quintile, only women in the most deprived quintile were more likely to experience delay in time to surgery > 31 days (OR 1.31; 95% CI: 1.17, 1.47). Key contributing factors to this reduction in OR were ethnicity and treatment facility type. A marginal but non-significant reduction in time to surgery was observed in the post-FCT period.

**Conclusion:**

Women residing in more deprived neighborhoods experienced greater delay in time to breast cancer surgery. Despite FCT implementation, urgent action is still needed to reduce inequities by deprivation in timely access to breast cancer surgery.

**Supplementary Information:**

The online version contains supplementary material available at 10.1007/s10552-025-02032-0.

## Background

Breast cancer is the most commonly diagnosed cancer among women in Aotearoa/New Zealand (NZ), with approximately 3,500 new cases each year, one of the highest incidence rates in OECD countries [1]. Breast cancer survival has improved over time in NZ [2] however it remains lower to other developed countries [3]. This is perpetuated by significant disparities in breast cancer survival rates—Māori, Pacific women, or those from lower socioeconomic backgrounds experience disproportionately poorer outcomes [4, 5].

One significant factor contributing to these disparities is socioeconomic deprivation, which has been shown to exacerbate delays in healthcare access and treatment [5, 6]. People living in more deprived areas often face barriers to accessing timely healthcare, leading to delays in diagnosis and treatment. Early detection and prompt treatment are crucial for improving outcomes. For early-stage breast cancer (stages 1–3a), surgery is the primary treatment, and delays in surgery have been shown to decrease survival rates [6–8]. Research has already highlighted that sociodemographic factors, including ethnicity, impact time to surgery, with Māori and Pacific women in NZ experiencing longer delays [9]. However, no studies have specifically investigated the relationship between socioeconomic deprivation and time to surgery.

In July 2012, the NZ Ministry of Health introduced the Faster Cancer Treatment (FCT) plan to facilitate equal and timely access to high-quality cancer care for *all* New Zealanders, irrespective of sociodemographic factors such as deprivation. This plan introduced the 31-day indicator which defined 31 days as the maximum acceptable delay from date of decision to treat to first cancer treatment [10]. To our knowledge, there have been no national studies evaluating the associations between deprivation and time to breast cancer surgery in NZ before or since FCT implementation.

This study assessed i. the association between neighbourhood deprivation and time to surgery in 20,322 women with early-stage (1–3a) breast cancer in NZ, from 2000 to 2020 and ii. whether these associations differed pre- and post- FCT implementation.

## Methods

### Study design and data sources

This retrospective study used the data from *Te Rēhita Mate Ūtaetae* (Breast Cancer Foundation National Register), a prospectively maintained database which recorded all primary breast cancer diagnoses in four large tertiary centres in NZ—Auckland, Waikato, Christchurch and Wellington. This comprises two-thirds of the country’s population and is representative of 63% of national breast cancer cases [2]. *Te Rēhita Mate Ūtaetae* is shown to be more comprehensive than national databases with detailed information on patient demographics, date and mode of diagnosis, tumour characteristics and treatment factors [11].

### Study population

**Fig. 1:**
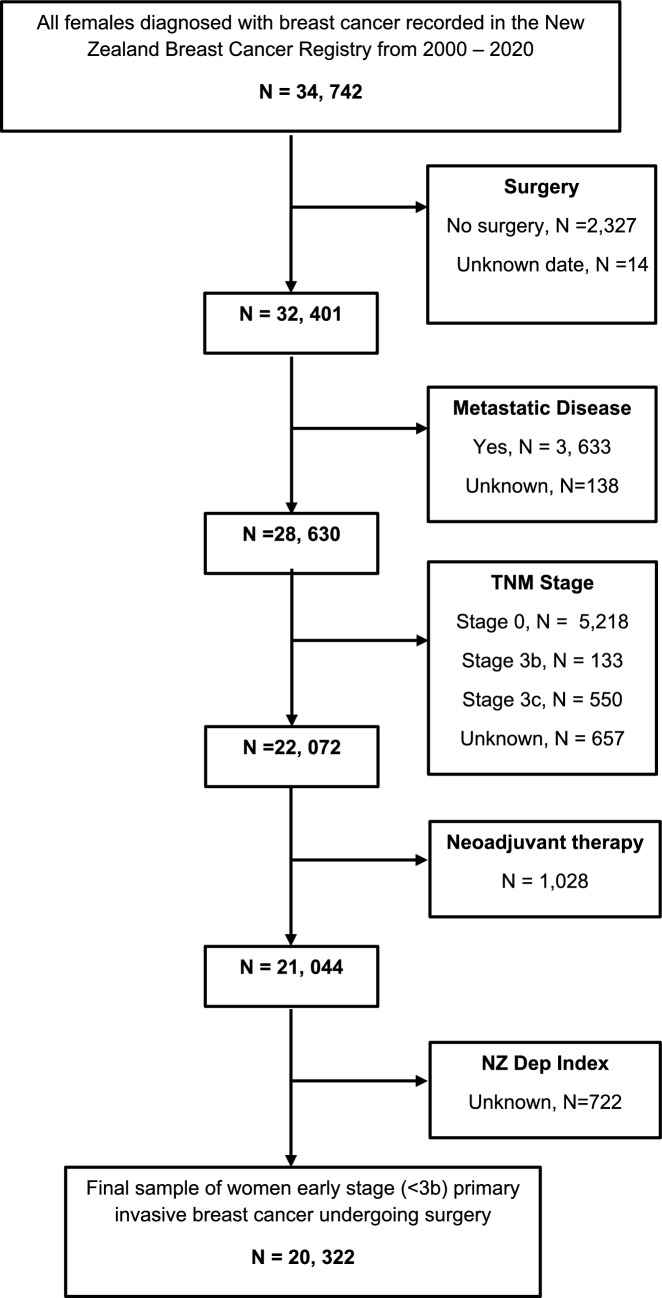
Sample restriction flowchart

This study included the 20,322 women who were diagnosed with histologically confirmed early-stage (1–3a) primary invasive breast cancer between 1 June 2000 and 31 December 2020 and received surgery as their primary cancer treatment. Women with metastases were excluded as these women do not have surgery as a primary treatment, as were those who did not undergo surgery (*n* = 2,327) or who had neoadjuvant treatment (*n* = 1,026) as this would affect time to surgery, as consistent with *Te Rēhita Mate Ūtaetae* research [2]. 721 women with missing NZ deprivation (NZDep) index were also excluded (Fig. 1).

### Variables of interest

The exposure of interest was deprivation measured with the NZDep index. This is an area-based measure of deprivation in NZ, based on nine census variables (e.g., unemployment, living in a rental property, access to a car—for all variables included see Supplementary Table S1), measured every national census. This is calculated in meshblocks which are the smallest geographical area defined by StatsNZ, with a population of around 60–110 people. It is measured in deciles, where 1 represents the least deprived and 10 represents the most deprived. NZDep01 (2001) was used for diagnoses between 2000 and 2005, NZDep06 (2006) for diagnoses between 2006 and 2010, NZDep13 for diagnoses between 2011 and 2015 and NZDep18 (2018) for diagnoses beyond 2015 [12]. This was categorised into quintiles: 1–2, 3–4, 5–6, 7–8 and 9–10.

The primary study outcome was time in days from date of diagnosis to date of surgery. Date of diagnosis was used as a proxy of ‘date of decision to treat’ per the FCT guideline. A threshold of 31 days was used as the limit for the longest acceptable delay in keeping with the FCT indicator set by the NZ Ministry of Health and time to surgery was categorised as a binary variable accordingly (≤ 31/ > 31 days) [10].

**Fig. 2:**
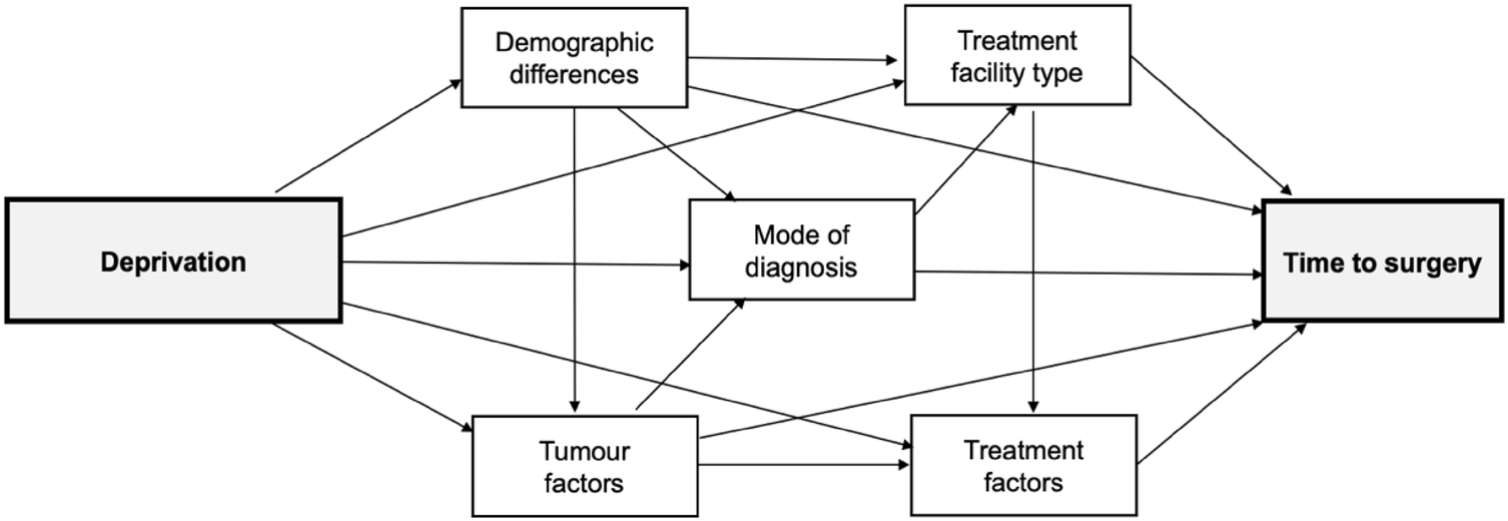
Conceptual framework displaying potential contributing factors on the deprivation-time to surgery association for women with breast cancer in Aotearoa/New Zealand

Other variables for analysis which may contribute to the association between ethnicity and type of surgery were selected a priori based on prior literature (Fig. 2) [4, 5, 7–9]. Those included in the models were: (1) demographic factors—age (< 45 years, ≥ 45 to ≤ 69 years (screening age in NZ), > 69 years), region, area of residence (rural/urban), ethnicity (NZ European, Māori, Asian, Pacific), (2) mode of diagnosis (screened/symptomatic which includes public and private), (3) tumour biology factors—TNM stage (1–3a)**,** grade (low, intermediate, high, unknown), histology (ductal, lobular, mixed, other, unknown), oestrogen and progesterone receptors (ER and PR) and human epidermal growth factor receptors (HER), (4) treatment facility for surgery (public/private) and (5) treatment factors—radiotherapy and systemic therapy (see Supplementary Table S2 for detail on variable categorization).

### Statistical analyses

Descriptive analyses summarized the data by NZDep index; the data were presented as proportions (%) and differences across NZDep quintiles were assessed using chi-squared (χ^2^) tests.

Using NZDep index 1–2 as the reference group, multivariable logistic regression models were built to obtain odds ratio (OR) with 95% confidence intervals (CI) for NZDep index and time to surgery. The models were adjusted in a step wise fashion, in five domains to build a total of five models for each outcome. Model one included adjustment for demographic factors, model two additionally included adjustment for mode of diagnosis, model three included adjustment for tumour factors, model four included adjustment for treatment facility and then the maximally adjusted model, model five, included adjustment for treatment factors. The linear trend was also tested by including NZDep categories as a continuous variable in the models.

Subgroup analyses by pre- and post FCT (using July 2012 as cut-off), mode of diagnosis and treatment facility type were undertaken. χ^2^ for heterogeneity were obtained to determine whether the risk estimates from these subgroup analyses were different. A *p*–value of 0.05 was considered statistically significant. The unadjusted and maximally adjusted models were also stratified by ethnicity.

Sensitivity analysis was undertaken with comorbidity added to maximally adjusted model, using the Charlson Comorbidity Index (CCI) by restricting the sample to the women with CCI recorded (*n* = 5,118) This is a validated index which measures the presence of up to 19 comorbidities and weights them according to their associated mortality risk to obtain a score, categorized as: 0, 1–2, 3–4 and ≥ 5 [13]. Data were analyzed in Stata MP version 17.0.

## Results

### Participant characteristics

Of the 20,322 women in the study, 4,777 (24%) were classified as NZDep 1–2 (least deprived), 4,519 (22%) were NZDep 3–4, 4,258 (21%) were NZDep 5–6, 3,957 (19%) were NZ 7–8 and 2,811 (14%) were NZDep 9–10 (most deprived group). The demographics, tumour and treatment factors for these women are displayed in Table 1. In terms of ethinicity there were 73.3% NZE women, 10.1% wāhine Māori, 6% Pacific women and 9.1% Asian women. Of note, the highest proportion of European women were in the NZDep 1–2 group (4,005), with the lowest proportion being in the NZDep 9–10 (1,247).

**Table 1:** Baseline demographics, tumour and treatment characteristics by NZ Deprivation

Characteristic, *n* (%)	Total (*n* = 20,322)	NZ Dep 1–2 (*n* = 4,777)	NZ Dep 3–4 (*n* = 4,519)	NZ Dep 5–6 (*n* = 4,258)	NZ Dep 7–8 (*n* = 3,957)	NZ Dep 9–10 (*n* = 2,811)	*P–value*^*a*^
Age (years)							
< 45	2,468 (12.1)	640 (13.4)	536 (11.9)	468 (11.0)	438 (11.1)	386 (13.7)	< 0.001
≥ 45 to ≤ 69	14,581 (71.8)	3,522 (73.7)	3,140 (69.5)	2,933 (68.9)	2,861 (72.3)	2,125 (75.6)	
> 69	3,273 (16.1)	615 (12.9)	843 (18.7)	857 (20.1)	658 (16.6)	300 (10.7)	
Ethnicity							
European	14,905 (73.3)	4,005 (83.8)	3,605 (79.8)	3,311 (77.7)	2,737 (69.2)	1,247 (44.4)	< 0.001
Māori	2,050 (10.1)	204 (4.3)	266 (5.9)	342 (8.0)	563 (14.2)	675 (24.0)	
Pacific	1,218 (6)	79 (1.7)	111 (2.5)	147 (3.5)	285 (7.2)	596 (21.2)	
Asian	1,847 (9.1)	420 (8.8)	466 (10.3)	396 (9.3)	315 (8.0)	250 (8.9)	
Other	302 (1.5)	69 (1.4)	71 (1.6)	62 (1.5)	57 (1.4)	43 (1.5)	
Region							
Auckland	11,607 (57.1)	2,674 (56.0)	2,818 (62.4)	2,391 (56.1)	2,009 (50.8)	1,715 (61.1)	< 0.001
Waikato	3,216 (15.8)	435 (9.1)	428 (9.5)	752 (17.7)	909 (23.0)	692 (24.6)	
Christchurch	2,975 (14.6)	949 (19.9)	745 (16.5)	571 (13.4)	557 (14.1)	153 (5.4)	
Wellington	2,524 (12.4)	719 (15.1)	528 (11.7)	544 (12.8)	482 (12.2)	251 (8.9)	
Area of residence							
Urban	18,244 (89.8)	4,172 (87.3)	3,867 (85.6)	3,780 (88.8)	3,714 (93.9)	2,711 (96.4)	< 0.001
Rural	2,078 (10.2)	605 (12.6)	652 (14.4)	478 (11.2)	243 (6.1)	100 (3.56)	
Mode of diagnosis							
Screen-detected	10,206 (50.2)	2,504 (52.4)	2,216 (49.0)	2,040 (47.9)	2,021 (51.1)	1,425 (50.7)	< 0.001
Symptomatic	10,116 (49.8)	2,273 (47.6)	2,303 (50.1)	2,218 (52.1)	1,936 (48.9)	1,386 (49.3)	
TNM Stage							
1a	10,719 (52.7)	2,587 (54.1)	2,420 (53.6)	2,245 (52.7)	2,081 (52.6)	1,386 (19.3)	< 0.001
1b	659 (3.2)	174 (3.6)	159 (3.5)	132 (3.1)	112 (2.8)	82 (2.9)	
2a	5,222 (25.7)	1,217 (25.5)	1,110 (24.6)	1,137 (26.7)	1,019 (25.8)	739 (26.3)	
2b	2,395 (11.8)	520 (10.9)	533 (11.8)	486 (11.4)	481 (12.2)	375 (13.3)	
3a	1,327 (6.4)	279 (5.8)	297 (6.6)	258 (6.1)	264 (6.7)	229 (8.2)	
Cancer grade							
Low	5,202 (25.6)	1,219 (25.5)	1,162 (25.7)	1,136 (26.7)	1,034 (26.1)	651 (23.2)	0.03
Intermediate	9,697 (47.7)	2,240 (46.9)	2,128 (47.1)	2,031 (47.7)	1,917 (48.5)	1,381 (49.1)	
High	5,218 (25.7)	1,268 (26.6)	1,184 (26.2)	1,041 (24.5)	976 (24.7)	749 (26.7)	
Unknown	205 (1.0)	50 (1.1)	45 (1.0)	50 (1.2)	30 (0.8)	30 (1.1)	
Histology							
Ductal	15,852 (78.0)	3,728 (78.0)	3,476 (76.9)	3,271 (76.8)	3,114 (78.7)	2,263 (80.5)	0.009
Lobular	2,451 (12.1)	588 (12.3)	568 (12.6)	559 (13.1)	466 (11.8)	270 (9.6)	
Mixed	589 (2.9)	126 (2.6)	152 (3.4)	114 (2.7)	113 (2.9)	84 (3.0)	
Other	1,231 (6.1)	283 (5.9)	278 (6.2)	268 (6.3)	232 (5.9)	170 (6.1)	
Unknown	198 (1.0)	52 (1.1)	44 (1.0)	46 (1.1)	32 (0.8)	24 (0.9)	
Receptors							
ER + /PR +	11,780 (58.0)	2,961 (62.0)	2,723 (60.3)	2,421 (56.9)	2,163 (54.7)	1,512 (53.8)	< 0.001
ER + /PR−	1,744 (8.6)	424 (8.9)	428 (9.5)	377 (8.9)	299 (7.6)	216 (7.7)	
ER−/PR +	169 (0.8)	41 (0.9)	42 (0.9)	31 (0.7)	33 (0.8)	22 (0.8)	
ER−/PR−	2,266 (11.2)	589 (12.3)	541 (12.0)	452 (10.6)	394 (10.0)	290 (10.3)	
Unknown	4,363 (21.5)	762 (16.0)	785 (17.4)	977 (23.0)	1,068 (27.0)	771 (27.4)	
HER							
Negative	15,650 (77.1)	3,754 (78.6)	3,528 (78.1)	3,280 (77.1)	2,988 (75.5)	2,100 (74.7)	< 0.001
Positive	2,468 (12.1)	568 (11.9)	531 (11.8)	460 (10.8)	502 (12.7)	407 (14.5)	
Unknown	2,204 (10.9)	455 (9.5)	460 (10.2)	518 (12.2)	467 (11.8)	304 (10.8)	
Treatment facility							
Private	7,225 (35.6)	2,455 (51.4)	1,943 (43.0)	1,474 (34.6)	969 (24.5)	384 (13.7)	< 0.001
Public	12,799 (63.0)	2,254 (47.2)	2,504 (55.4)	2,725 (64.0)	2,921 (73.8)	2,395 (85.2)	
Unknown	298 (1.5)	68 (1.4)	72 (1.6)	59 (1.4)	67 (1.7)	32 (1.1)	
Locoregional treatment							
BCS + RT	9,676 (47.6)	2,283 (47.8)	2,098 (46.4)	2,047 (48.1)	1,903 (48.1)	1,345 (47.9)	< 0.001
BCS no RT	1,751 (8.6)	419 (8.8)	375 (8.3)	395 (9.3)	322 (8.1)	240 (8.5)	
Mastectomy	7,909 (38.9)	1,777 (37.2)	1,813 (40.1)	1,622 (38.1)	1,534 (38.8)	1,163 (41.4)	
Unknown	986 (4.9)	298 (6.2)	233 (5.2)	194 (4.6)	198 (5.0)	63 (2.2)	
Systemic therapy							
Yes	14,888 (73.3)	3,473 (72.7)	3,204 (70.9)	3,081 (72.4)	2,971 (75.1)	2,159 (76.8)	< 0.001
No	5,434 (26.7)	1,304 (27.3)	1,315 (29.1)	1,177 (27.6)	986 (24.9)	652 (23.2)	

^*a*^*p* value for *χ*^2^ tests

*NZ Dep* New Zealand Deprivation, *TNM* Tumour Node Metastasis Stage, *ER* Oestrogen Receptor, *PR* Progesterone Receptor, *HER* Human Epidermal Growth Factor Receptor, *BCS* Breast Conserving Surgery, *RT* Radiotherapy

### Time to surgery

Fifty–seven percent (n = 11,524 /20,322) of all women received surgery in 31 days. In unadjusted models, compared to women in the least deprived quintile (NZDep 1–2) women in all other quintiles were more likely to experience delay to surgery > 31 days with the greatest delay experienced in the two most deprived quintiles (NZDep 7–8 OR 1.72; 95% CI: 1.58,1.87 and NZDep 9–10 OR 2.56; 95% CI 2.32, 2.81). In maximally adjusted models, compared to women in the least deprived quintile (NZDep 1–2), women in the most deprived quintile (NZDep 9–10) remained 31% more likely to experience delay > 31 days (OR 1.31; 95% CI 1.17,1.47). Key factors contributing to the attenuation in OR for all quintiles were ethnicity and treatment facility type (Table 2). The linear test for trend was significant for both unadjusted and adjusted models (*p* < 0.001 and *p* = 0.001, respectively).

**Table 2:** Multivariate logistic regression models displaying odds ratios (with 95% confidence intervals) for time to surgery > 31 days versus ≤ 31 days by deprivation

Model	Additional variables in model^a^	NZ Dep 1–2 (Reference) (*n* = 4,777) (*n* = 1,681 for surgery > 31 days)	NZ Dep 3–4 (*n* = 4,519) (*n* = 1,775 for surgery > 31 days)	NZ Dep 5–6 (*n* = 4,258) (*n* = 1,797 for surgery > 31 days)	NZ Dep 7–8 (*n* = 3,957) (*n* = 1,911 for surgery > 31 days)	NZ Dep 9–10 (*n* = 2,811) (*n* = 1,634 for surgery > 31 days)
Unadjusted		1.0	1.19 (1.10–1.30)	1.34 (1.24–1.46)	1.72 (1.58–1.87)	2.56 (2.32–2.81)
1. Unadjusted + Demographics	Age	1.0	1.17 (1.08–1.28)	1.32 (1.21–1.44)	1.70 (1.56–1.85)	2.59 (2.35–2.85)
	Ethnicity	1.0	1.15 (1.06–1.26)	1.27 (1.17–1.39)	1.56 (1.43–1.71)	2.10 (1.90–2.33)
	Region	1.0	1.18 (1.09–1.28)	1.26 (1.16–1.37)	1.50 (1.38–1.64)	2.05 (1.85–2.27)
	Area of residence	1.0	1.18 (1.08–1.28)	1.26 (1.16–1.37)	1.50 (1.37–1.64)	2.05 (1.84–2.27)
2. Model 1 + Mode of diagnosis	Mode of diagnosis	1.0	1.19 (1.09–1.29)	1.28 (1.17–1.39)	1.51 (1.38–1.65)	2.08 (1.87-2.30)
3. Model 2 + Tumour factors	Stage	1.0	1.19 (1.09–1.29)	1.28 (1.17–1.39)	1.51 (1.38–1.65)	2.08 (1.87–2.30)
	Grade	1.0	1.19 (1.09–1.29)	1.27 (1.17–1.39)	1.51 (1.38–1.66)	2.09 (1.88–2.32)
	Histology	1.0	1.19 (1.09–1.30)	1.27 (1.17–1.39)	1.52 (1.39–1.66)	2.11 (1.90–2.34)
	ER/PR	1.0	1.19 (1.09–1.29)	1.27 (1.17–1.39)	1.52 (1.38–1.66)	2.12 (1.91–2.35)
	HER	1.0	1.19 (1.09–1.30)	1.28 (1.17–1.40)	1.53 (1.40–1.67)	2.13 (1.92–2.37)
4. Model 3 + Treatment facility	Treatment facility	1.0	1.06 (0.96–1.16)	1.00 (0.91–1.10)	1.06 (0.96–1.16)	1.33 (1.19-1.49)
5. Model 4 + Treatment factors	Locoregional	1.0	1.05 (0.96–1.16)	1.00 (0.91–1.10)	1.05 (0.95–1.16)	1.32 (1.17–1.47)
	Systemic	1.0	1.05 (0.96–1.16)	1.00 (0.91–1.10)	1.05 (0.95–1.16)	1.31 (1.17–1.47)

^a^Variables are categorized as follows: age; < 45 years, ≥ 45 to ≤ 69 years (women eligible for BSA) and > 69 years, ethnicity; NZ European, Asian, Māori, Pacific, region; Auckland, Waikato, Wellington Christchurch, area of residence; rural or urban, mode of diagnosis; screened or symptomatic, stage; using AJCC 7th edition TNM staging, grade; 1—low to 3—high, histology; ductal, lobular, mixed, other, ER/PR; ER + /PR + , ER + /PR−, ER−/PR + , ER−/PR−, unknown, HER; negative, equivocal, positive, unknown, treatment facility for surgery; public or private, locoregional; BCS with radiotherapy, BCS without radiotherapy, mastectomy, systemic; systemic treatment(chemotherapy, hormone therapy or biologics) or no systemic treatment

### Subgroup analyses by pre- and post- FCT implementation

Compared to women in the least deprived quintile (NZDep 1–2) women in the most deprived quintile (NZDep 9–10), were more likely to experience delay to surgery > 31 days in both the pre- and post FCT periods (OR 1.42; 95% CI:1.17, 1.71 and OR 1.28; 95% CI: 1.11, 1.49, respectively). However, this difference in the associations in the pre- vs post- FCT period for the most deprived group was non-significant (*p*_heterogeneity_ = 0.43). The OR for other quintiles was also not significant (Table 3).

**Table 3:** Subgroup analysis multivariate logistic regression models displaying odds ratios (with 95% confidence intervals) for time to surgery > 31 days versus ≤ 31 days by NZ deprivation index before and after implementation of Faster Cancer Treatment targets

Model	Additional variables in model^c^	Pre-FCT^a^ OR^b^ (*n* = 8,343)				Post-FCT^a^ OR^b^ (*n* = 11,979)			
		NZDep 3–4 (*n* = 1,808) (*n* = 1,681 for surgery > 31 days)	NZDep 5–6 (*n* = 1,820) (*n* = 1,681 for surgery > 31 days)	NZDep 7–8 (*n* = 1,711) (*n* = 1,681 for surgery > 31 days)	NZDep 9–10 (*n* = 1,172) (*n* = 1,681 for surgery > 31 days)	NZDep 3–4 (*n* = 2,711) (*n* = 1,681 for surgery > 31 days)	NZDep 5–6 (*n* = 2,438) (*n* = 1,681 for surgery > 31 days)	NZDep 7–8 (*n* = 2,246) (*n* = 1,681 for surgery > 31 days)	NZDep 9–10 (*n* = 1,639) (*n* = 1,681 for surgery > 31 days)
Unadjusted		1.16 (1.00,1.34)	1.56 (1.35, 1.80)	1.92 (1.66, 2.21)	2.85 (2.44, 3.34)	1.25 (1.12, 1.38)	1.31 (1.18, 1.46)	1.75 (1.57, 1.96)	2.60 (2.30, 2.95)
1. Unadjusted + Demographics	Age	1.13 (0.98,1.31)	1.50 (1.30, 1.74)	1.86 (1.61, 2.16)	2.88 (2.46, 3.37)	1.24 (1.11, 1.37)	1.30 (1.17, 1.45)	1.75 (1.56, 1.95)	2.62 (2.31, 2.98)
	Ethnicity	1.11 (0.96, 1.29)	1.45 (1.25, 1.68)	1.71 (1.47, 1.98)	2.33 (1.97, 2.74)	1.22 (1.10, 1.36)	1.27 (1.14, 1.41)	1.63 (1.45, 1.82)	2.20 (1.93, 2.51)
	Region	1.12 (0.96, 1.30)	1.39 (1.20, 1.61)	1.60 (1.38, 1.85)	2.19 (1.85, 2.58)	1.22 (1.10, 1.36)	1.24 (1.11, 1.40)	1.54 (1.37, 1.72)	2.05 (1.27, 1.72)
	Area of residence	1.13 (0.97, 1.31)	1.39 (1.20, 1.61)	1.58 (1.36, 1.84)	2.16 (1.83, 2.55)	1.22 (1.10, 1.36)	1.24 (1.11, 1.39)	1.54 (1.37, 1.73)	2.06 (1.80, 2.36)
2. Model 1 + Mode of diagnosis	Mode of diagnosis	1.14 (0.98, 1.33)	1.41 (1.22, 1.64)	1.59 (1.37, 1.85)	2.22 (1.88, 2.63)	1.23 (1.10, 1.37)	1.25 (1.12, 1.40)	1.55 (1.38, 1.74)	2.08 (1.81, 2.38)
3. Model 2 + Tumour factors	Stage	1.14 (0.98, 1.33)	1.41 (1.22, 1.64)	1.59 (1.37, 1.84)	2.22 (1.87, 2.62)	1.23 (1.10, 1.37)	1.25 (1.12, 1.40)	1.55 (1.38, 1.74)	2.08 (1.81, 2.38)
	Grade	1.14 (0.98, 1.32)	1.40 (1.21, 1.63)	1.59 (1.36, 1.84)	2.23 (1.89, 2.64)	1.24 (1.11, 1.38)	1.25 (1.12, 1.40)	1.56 (1.39, 1.75)	2.09 (1.82, 2.40)
	Histology	1.14 (0.98, 1.32)	1.40 (1.21, 1.63)	1.59 (1.37, 1.85)	2.24 (1.90, 2.66)	1.24 (1.11, 1.38)	1.25 (1.12, 1.40)	1.56 (1.39, 1.75)	2.11 (1.84, 2.42)
	ER/PR	1.14 (0.98, 1.32)	1.40 (1.21, 1.83)	1.58 (1.36, 1.83)	2.24 (1.89, 2.65)	1.24 (1.11, 1.38)	1.25 (1.12, 1.40)	1.56 (1.39, 1.75)	2.11 (1.84, 2.43)
	HER	1.14 (0.98, 1.33)	1.41 (1.22, 1.64)	1.60 (1.37, 1.86)	2.27 (1.91, 2.69)	1.24 (1,11, 1.38)	1.25 (1.12, 1.40)	1.56 (1.39, 1.76)	2.11 (1.84, 2.42)
4. Model 3 + Treatment facility	Treatment facility	1.02 (0.85, 1.21)	1.09 (0.93, 1.29)	1.13 (0.95, 1.33)	1.41 (1.17, 1.70)	1.11 (0.99, 1.25)	1.00 (0.88, 1.12)	1.07 (0.94, 1.21)	1.32 (1.13, 1.53)
5. Model 4 + Treatment factors	Locoregional	1.03 (0.87, 1.22)	1.10 (0.93, 1.30)	1.14 (0.96, 1.35)	1.42 (1.17, 1.71)	1.10 (0.98, 1.24)	0.99 (0.88, 1.12)	1.05 (0.93, 1.19)	1.29 (1.12, 1.50)
	Systemic	1.04 (0.87, 1.23)	1.10 (0.94, 1.30)	1.15 (0.97, 1.36)	1.42 (1.17, 1.71)	1.10 (0.97, 1.23)	0.99 (0.88, 1.11)	1.05 (0.93, 1.19)	1.28 (1.11, 1.49)

^a^1 day Faster Cancer Treatment indicators implemented 01 July 2012 by the Ministry of Health, pre-FCT includes patients diagnosed before 01 July 2012, post-FCT includes patients diagnosed on or after 01 July 2012

^b^OR using NZDep 1–2 at reference group

c.Variables are categorized as follows: age; < 45 years, ≥ 45 to ≤ 69 years (women eligible for BSA) and > 69 years, ethnicity; NZ European, Asian, Māori, Pacific, region; Auckland, Waikato, Wellington Christchurch, area of residence; rural or urban, mode of diagnosis; screened or symptomatic, stage; using AJCC 7th edition TNM staging, grade; 1—low to 3—high, histology; ductal, lobular, mixed, other, ER/PR; ER + /PR + , ER + /PR−, ER−/PR + , ER−/PR−, unknown, HER; negative, equivocal, positive, unknown, treatment facility for surgery; public or private, locoregional; BCS with radiotherapy, BCS without radiotherapy, mastectomy, systemic; systemic treatment (chemotherapy, hormone therapy or biologics) or no systemic treatment

### Subgroup analyses by treatment facility type

Almost two-thirds of patients (63%, n = 12,799) were treated in the public sector. When analyzed by treatment facility type, when compared to women in NZDep 1–2, women in NZDep 9–10 were more likely to experience delay in the public sector only (OR 1.34; 95% CI: 1.18, 1.52), with no significant difference in the private sector (OR 1.20; 95% CI: 0.90, 1.62). Additionally, there were no significant delays experienced for any deprivation quintile in the private sector (Table 4).

**Table 4:** Subgroup analysis multivariate logistic regression models displaying odds ratios (with 95% confidence intervals) for time to surgery > 31 days versus ≤ 31 days by NZ deprivation index by treatment facility type

Model	Additional variables in model^b^	Public OR^a^ (*n* = 12,799)				Private OR^a^ (*n* = 7,225)			
		NZDep 3–4 (*n* = 2,504) (*n* = 1,681 for surgery > 31 days)	NZDep 5–6 (*n* = 2,725) (*n* = 1,681 for surgery > 31 days)	NZDep 7–8 (*n* = 2,921) (*n* = 1,681 for surgery > 31 days)	NZDep 9–10 (*n* = 2, 395) (*n* = 1,681 for surgery > 31 days)	NZDep 3–4 (*n* = 1,943) (*n* = 1,681 for surgery > 31 days)	NZDep 5–6 (*n* = 1,474) (*n* = 1,681 for surgery > 31 days)	NZDep 7–8 (*n* = 969) (*n* = 1,681 for surgery > 31 days)	NZDep 9–10 (*n* = 384) (*n* = 1,681 for surgery > 31 days)
Unadjusted		1.11 (1.00, 1.25)	1.08 (0.96, 1.20)	1.23 (1.10, 1.37)	1.54 (1.36, 1.73)	0.94 (0.80, 1.10)	0.91 (0.77, 1.09)	0.90 (0.74, 1.10)	1.09 (0.83, 1.43)
1. Unadjusted + Demographics	Age	1.12 (1.00, 1.26)	1.08 (0.97, 1.21)	1.22 (1.09, 1.37)	1.55 (1.38, 1.75)	0.93 (0.80, 1.09)	0.91 (0.76, 1.08)	0.90 (0.74, 1.10)	1.09 (0.83, 1.44)
	Ethnicity	1.12 (1.00, 1.25)	1.07 (0.95, 1.19)	1.19 (1.06, 1.33)	1.46 (1.29, 1.65)	0.93 (0.80, 1.09)	0.90 (0.76, 1.07)	0.88 (0.72, 1.08)	1.04 (0.79, 1.38)
	Region	1.11 (0.99, 1.25)	1.03 (0.92, 1.15)	1.13 (1.00, 1.26)	1.35 (1.19, 1.53)	1.01 (0.86, 1.19)	0.93 (0.78, 1.11)	0.91 (0.74, 1.12)	1.11 (0.83, 1.48)
	Area of residence	1.11 (0.99, 1.25)	1.03 (0.92, 1.15)	1.12 (1.00, 1.51)	1.34 (1.18, 1.52)	1.02 (0.86, 1.19)	0.93 (0.78, 1.11)	0.91 (0.74, 1.11)	1.10 (0.82, 1.46)
2. Model 1 + Mode of diagnosis	Mode of diagnosis	1.19 (1.00, 1.26)	1.04 (0.92, 1.16)	1.12 (1.00, 1.26)	1.35 (1.19, 1.54)	1.02 (0.87, 1.20)	0.94 (0.78, 1.11)	0.91 (0.74, 1.11)	1.12 (0.84, 1.50)
3. Model 2 + Tumour factors	Stage	1.12 (1.00, 1.26)	1.04 (0.93, 1.17)	1.12 (1.00, 1.26)	1.35 (1.19, 1.54)	1.02 (0.87, 1.20)	0.93 (0.79, 1.12)	0.91 (0.74, 1.11)	1.12 (0.81, 1.50)
	Grade	1.12 (1.00, 1.26)	1.03 (0.92, 1.16)	1.13 (1.00, 1.26)	1.36 (1.20, 1.55)	1.02 (0.87, 1.20)	0.93 (0.78, 1.11)	0.90 (0.78, 1.11)	1.13 (0.85, 1.51)
	Histology	1.12 (1.00, 1.26)	1.03 (0.92, 1.16)	1.13 (1.00, 1.27)	1.37 (1.21, 1.56)	1.02 (0.87, 1.20)	0.93 (0.78, 1.11)	0.90 (0.73, 1.11)	1.13 (0.84, 1.50)
	ER/PR	1.12 (1.00, 1.26)	1.03 (0.92, 1.16)	1.13 (1.00, 1.26)	1.37 (1.21, 1.56)	1.02 (0.87, 1.20)	0.93 (0.78, 1.12)	0.91 (0.74, 1.11)	1.14 (0.85, 1.53)
	HER	1.12 (1.00, 1.26)	1.04 (0.92, 1.16)	1.13 (1.00, 1.27)	1.38 (1.21, 1.57)	1.02 (0.86, 1.20)	0.93 (0.78, 1.12)	0.92 (0.75, 1.13)	1.16 (0.87, 1.56)
4. Model 3 + Treatment factors	Locoregional	1.11 (0.99, 1.25)	1.03 (0.92, 1.16)	1.11 (0.99, 1.25)	1.35 (1.19, 1.54)	1.02 (0.86, 1.20)	0.95 (0.80, 1.14)	0.94 (0.76, 1.17)	1.20 (0.90, 1.61)
	Systemic	1.11 (0.98, 1.25)	1.03 (0.91, 1.15)	1.11 (0.99, 1.25)	1.34 (1.18, 1.52)	1.01 (0.86, 1.20)	0.95 (0.80, 1.14)	0.95 (0.77, 1.17)	1.20. (0.90, 1.62)

^a^OR using NZDep 1–2 at reference group

^b^Variables are categorized as follows: age; < 45 years, ≥ 45 to ≤ 69 years (women eligible for BSA) and > 69 years, ethnicity; NZ European, Asian, Māori, Pacific, region; Auckland, Waikato, Wellington Christchurch, area of residence; rural or urban, mode of diagnosis; screened or symptomatic, stage; using AJCC 7th edition TNM staging, grade; 1—low to 3—high, histology; ductal, lobular, mixed, other, ER/PR; ER + /PR + , ER + /PR−, ER−/PR + , ER−/PR−, unknown, HER; negative, equivocal, positive, unknown, treatment facility for surgery; public or private, locoregional; BCS with radiotherapy, BCS without radiotherapy, mastectomy, systemic; systemic treatment (chemotherapy, hormone therapy or biologics) or no systemic treatment

### Subgroup analyses by mode of diagnosis

Half (50.2%) of the breast cancers were diagnosed through screening. There were similar patterns between the deprivation and time to surgery association seen in the screened and symptomatic groups (Table 5).

**Table 5:** Subgroup analysis multivariate logistic regression models displaying odds ratios (with 95% confidence intervals) for time to surgery > 31 days versus ≤ 31 days by NZ deprivation index by mode of diagnosis

Model	Additional variables in model^b^	Screened OR^a^ (*n* = 10,206)				Symptomatic OR^a^ (*n* = 10,116)			
		NZDep 3–4 (*n* = 2,216) (*n* = 1,681 for surgery > 31 days)	NZDep 5–6 (*n* = 2,040) (*n* = 1,681 for surgery > 31 days)	NZDep 7–8 (*n* = 2,021) (*n* = 1,681 for surgery > 31 days)	NZDep 9–10 (*n* = 1,425) (*n* = 1,681 for surgery > 31 days)	NZDep 3–4 (*n* = 2,303) (*n* = 1,681 for surgery > 31 days)	NZDep 5–6 (*n* = 2,218) (*n* = 1,681 for surgery > 31 days)	NZDep 7–8 (*n* = 1,936) (*n* = 1,681 for surgery > 31 days)	NZDep 9–10 (*n* = 1,386) (*n* = 1,681 for surgery > 31 days)
Unadjusted		1.20 (1.07, 1.35)	1.30 (1.15, 1.46)	1.63 (0.95, 1.38)	2.26 (1.83, 2.79)	1.22 (1.08, 1.38)	1.45 (1.28, 1.64)	1.86 (1.64, 2.11)	2.14 (1.86, 2.45)
1. Unadjusted + Demographics	Age	1.20 (1.07, 1.35)	1.29 (1.15, 1.45)	1.61 (1.43, 1.81)	3.11(2.72, 3.58)	1.15 (1.01, 1.30)	1.34 (1.16, 1.49)	1.77 (1.56, 2.00)	2.16 (1.89, 2.48)
	Ethnicity	1.17 (1.04, 1.31)	1.24 (1.10, 1.61)	1.43 (1.26, 1.70)	2.31 (2.00, 2.67)	1.13 (1.00, 1.28)	1.31 (1.57, 2.09)	1.69 (1.49, 1.92)	1.96 (1.69, 2.27)
	Region	1.17 (1.04, 1.31)	1.22 (1.08, 1.37)	1.37 (1.21, 1.55)	2.20 (1.90, 2.55)	1.19 (1.05, 1.36)	1.32 (1.16, 1.50)	1.63 (1.43, 1.86)	1.98 (1.70, 2.31)
	Area of residence	1.17 (1.04, 1.31)	1.22 (1.08, 1.37)	1.37 (1.21, 1.54)	2.20 (1.90, 2.55)	1.19 (1.05, 1.36)	1.32 (1.16, 1.50)	1.63 (1.43, 1.86)	1.98 (1.70, 2.31)
2. Model 1 + Tumour factors	Stage	1.17 (1.04, 1.31)	1.22 (1.08, 1.37)	1.37 (1.22, 1.56)	2.21 (1.90, 2.55)	1.19 (1.05, 1.36)	1.32 (1.16, 1.50)	1.64 (1.43, 1.87)	1.99 (1.71, 2.31)
	Grade	1.17 (1.04, 1.31)	1.22 (1.08, 1.37)	1.37 (1.22, 1.55)	2.22 (1.91, 2.57)	1.20 (1.16, 1.50)	1.32 (1.16, 1.50)	1.64 (1.44, 1.87)	2.01 (1.72, 2.34)
	Histology	1.17 (1.04, 1.31)	1.21 (1.07, 1.36)	1.38 (1.22, 1.56)	2.24 (1.93, 2.59)	1.19 (1.05, 1.36)	1.32 (1.16, 1.50)	1.65 (1.44, 1.87)	2.02 (1.73, 2.35)
	ER/PR	1.17 (1.04, 1.32)	1.21 (1.08, 1.37)	1.38 (1.22, 1.56)	2.25 (1.94, 2.61)	1.20 (1.05, 1.36)	1.32 (1.16, 1.50)	1.64 (1.44, 1.87)	2.03 (1.74, 2.37)
	HER	1.17 (1.04, 1.32)	1.22 (1.08, 1.38)	1.39 (1.23, 1.58)	2.27 (1.96, 2.64)	1.20 (1.06, 1.36)	1.33 (1.17, 1.51)	1.65 (1.45, 1.89)	2.03 (1.74, 2.37)
3. Model 2 + Treatment facility	Treatment facility	1.05 (0.92, 1.20)	0.96 (0.84, 1.10)	0.94 (0.82, 1.08)	1.40 (1.19, 1.65)	1.07 (0.93, 1.23)	1.05 (0.91, 1.21)	1.19 (1.03, 1.37)	1.32 (1.12, 1.55)
4. Model 3 + Treatment factors	Locoregional	1.05 (0.92, 1.20)	0.97 (0.85, 1.11)	0.94 (0.82, 1.07)	1.39 (1.18, 1.63)	1.07 (0.93, 1.23)	1.05 (0.91, 1.20)	1.19 (1.03, 1.37)	1.31 (1.11, 1.55)
	Systemic	1.05 (0.92, 1.20)	0.97 (0.84, 1.10)	0.94 (0.82, 1.07)	1.38 (1.18, 1.63)	1.06 (0.92, 1.22)	1.04 (0.90, 1.20)	1.19 (1.03, 1.37)	1.21. (1.10, 1.53)

^a^OR using NZDep 1–2 at reference group

^b^Variables are categorized as follows: age; < 45 years, ≥ 45 to ≤ 69 years (women eligible for BSA) and > 69 years, ethnicity; NZ European, Asian, Māori, Pacific, region; Auckland, Waikato, Wellington Christchurch, area of residence; rural or urban, mode of diagnosis; screened or symptomatic, stage; using AJCC 7th edition TNM staging, grade; 1—low to 3—high, histology; ductal, lobular, mixed, other, ER/PR; ER + /PR + , ER + /PR−, ER−/PR + , ER−/PR−, unknown, HER; negative, equivocal, positive, unknown, treatment facility for surgery; public or private, locoregional; BCS with radiotherapy, BCS without radiotherapy, mastectomy, systemic; systemic treatment (chemotherapy, hormone therapy or biologics) or no systemic treatment

### Analysis stratified by ethnicity

When stratified by ethnicity, in unadjusted models, Asian, NZE and Māori women in the most deprived group experienced a delay in surgery > 31 days, compared to those within the least deprived groups (OR for NZE women 2.20; 95% 1.93, 2.51, OR for Māori women 2.58; CI: 1.88, 3.55 and OR for Asian women 1.95; 95% CI: 1.41, 2.69). In maximally adjusted models, this difference only persisted for NZE and Asian women (OR 2.19; 95% CI: 1.93, 2.50 and OR1.62; 95% CI:1.12, 2.34, respectively) (Table 6).

**Table 6:** Unadjusted and adjusted multivariate logistic regression models displaying odds ratios (with 95% confidence intervals) for time to surgery > 31 days versus ≤ 31 days stratified by ethnicity

	NZ Dep 1–2 (Reference)	NZ Dep 3–4 (*n* = 4,519)	NZ Dep 5–6 (*n* = 4,258)	NZ Dep 7–8 (*n* = 3,957)	NZ Dep 9–10 (*n* = 2,811)
Number of people within each NZ Dep index (n)					
NZ European (*n* = 14,905)	4,005	3,605	3,311	2,737	1,247
Māori	204	266	342	563	675
Pacific	79	111	147	285	596
Asian	420	466	396	315	250
Unadjusted OR (95% CI) for time to surgery by ethnicity					
NZ European	1.0	1.17 (1.06, 1.28)	1.31 (1.20, 1.45)	1.60 (1.45, 1.77)	2.20 (1.93, 2.50)
Māori	1.0	1.16 (0.80, 1.67)	1.49 (1.05, 2.12)	1.85 (1.34, 2.56)	2.58 (1.88. 3.55)
Pacific	1.0	0.79 (0.44, 1.41)	1.25 (0.72, 2.17)	1.15 (0.70, 1.90)	1.41 (0.88, 2.26)
Asian	1.0	1.33 (1.02, 1.74)	1.08 (0.81, 1.43)	1.48 (1.10, 1.99)	1.95 (1.42, 2.69)
Maximally adjusted^a^ OR (95% CI) for time to surgery by ethnicity					
NZ European	1.0	1.04 (0.94–1.16)	0.98 (0.88–1.10)	1.04 (0.93–1.16)	1.27 (1.10–1.48)
Māori	1.0	0.81 (0.53–1.23)	0.90 (0.60–1.34)	0.98 (0.67–1.43)	1.39 (0.95–2.02)
Pacific	1.0	0.61 (0.31–1.20)	1.20 (0.63–2.28)	0.86 (0.48–1.55)	1.05 (0.60–1.84)
Asian	1.0	1.48 (1.08–2.01)	1.07 (0.78–1.48)	1.23 (0.88–1.72)	1.67 (1.16–2.41)

^a^Maximally adjusted model includes adjustment for (1) demographic factors—age (< 45 years, ≥ 45 to ≤ 69 years (screening age in NZ), > 69 years), region, area of residence (rural/urban), (2) mode of diagnosis (screened/symptomatic which includes public and private), (3) tumour biology factors—TNM stage (1–3a), grade (low, intermediate, high, unknown), histology (ductal, lobular, mixed, other, unknown), oestrogen and progesterone receptors (ER and PR) and human epidermal growth factor receptors (HER), (4) treatment facility for surgery (public/private) and (5) treatment factors—radiotherapy and systemic therapy

Note 302 people of other ethnicity were excluded

### Sensitivity analysis

CCI was recorded for 5,118 women. 72.8% had a score of 0, 23.9% a score of 1–2, 2.2% a score of 3–4 and 1.1% a score ≥ 5. Inclusion of CCI in the model did not significantly alter the OR for time to surgery for any NZDep category compared to NZDep 1–2 (supplementary Table S3).

## Discussion

In this study involving women diagnosed with early-stage breast cancer in NZ between 2000 and 2020, just over half of women (57%) received surgery within 31 days. Women in the most deprived group were more likely to experience delay in time to surgery, when compared to women in the least deprived group. Increasing deprivation was associated with increasing odds of delay > 31 days, which was mainly contributed by ethnicity and treatment facility type.

Almost one in two women with early-stage breast cancer did not receive surgery within the recommended 31-day target. This falls well below the NZ national recording in December 2022 of 88% (all cancer types), and also well below the UK recording in April 2021 of 94% (all cancer types) [14, 15]. This target was introduced in the FCT plan in 2012, to ensure timely and equitable access to cancer treatments [10]. This study highlights that significant inequities still exist as we only observed a minor and non-significant reduction in the OR in the post-FCT group. By separating the target by breast cancer stream and deprivation level, this study demonstrates that *significant* additional efforts are needed to achieve the 31-day target for women with breast cancer. This supports the finding from our prior paper, which separated the target by ethnicity, and found delay in time to surgery for Māori and Pacific women in the post-FCT period [9]. These two studies demonstrate that, despite almost 13 years since the FCT plan was introduced, disparities persist, underscoring the urgent need for targeted interventions to ensure timely, equitable access to breast cancer care for all women, regardless of ethnicity and deprivation.

The findings from this study align with previous research that suggests socioeconomic factors, including deprivation, significantly impact timely access to breast cancer treatment. Prior studies in other medical domains have shown that women from lower socioeconomic backgrounds experience poorer access to healthcare, which may be attributed to barriers such as access to care, healthcare provider availability, and financial constraints [16–20]. In NZ, deprivation is a key determinant of health outcomes, with individuals from more deprived areas experiencing poorer access to healthcare services. Those from lower socioeconomic backgrounds, often Māori and Pacific communities, are more likely to experience delays due to structural inequities in the healthcare system. These delays are compounded by both socioeconomic factors and the legacy of colonization, which has created disparities in access to health services [8, 21, 22]. However, it is interesting that when the results were stratified by ethnicity, in maximally adjusted models, only NZE and Asian women from the most deprived group experienced delay in time to surgery compared to the least deprived group—Māori and Pacific women did not. This parallels a previously established finding that breast cancer survival does not vary by deprivation index for Māori wāhine but does for NZE women [20].

This study uniquely highlights the role of both ethnicity and treatment facility type as contributing factors, which prior studies have not fully explored. While some literature has noted the impact of treatment facility infrastructure, particularly between public and private facilities [9, 21], the present study’s finding that women in the most deprived group only experienced delays within the public sector provides new insight into the differential access and potential inequities within different care settings, This observation is particularly relevant in the NZ context, where public healthcare facilities, which serve a higher proportion of socioeconomically disadvantaged populations, are often overburdened with longer wait times and resource limitations [18, 23]. In contrast, those with the financial means to access private care are typically able to bypass these delays. This raises important questions about resource allocation and the need for targeted interventions in public healthcare settings to mitigate these delays and address inequities in access to timely care.

The findings from this study underscore the intersectional nature of healthcare delays, with both ethnicity and treatment facility type playing pivotal roles in shaping timely access to surgery, as demonstrated previously. Women from Māori and Pacific ethnic backgrounds, particularly those from the most deprived groups, experienced disproportionately higher delays in receiving surgery, a trend also linked to the type of healthcare facility they accessed [9]. It is important to acknowledge that these findings reflect the downstream effects of colonization. Previous research has acknowledged the challenges faced by minority ethnic groups in NZ, where healthcare disparities are often compounded by structural inequities, cultural differences, and implicit biases [8, 22]. These delays are particularly pronounced in public treatment settings, which tend to serve a higher proportion of socioeconomically disadvantaged populations, a group who are unlikely to have access to healthcare insurance, which funds treatment in the private sector. Public hospitals, compared to private ones, face resource limitations, longer wait times, and higher patient volumes, which can exacerbate delays in care [24, 25]. This study uniquely contributes to the literature by revealing the compounded disadvantage experienced by women in public hospitals, who are also more likely to be Māori, Pacific, or from lower socioeconomic backgrounds. Therefore, it is clear that addressing these disparities requires multifaceted interventions, not only focusing on ethnicity and socioeconomic status but also on structural changes within the healthcare system to ensure equitable care across all facilities.

These findings therefore prompt urgent targeted policy reforms and resource allocation aimed at reducing delays and improving equity in breast cancer care, particularly within the public sector. Although we were unable to pinpoint all the causes, our findings can be used to guide potential interventions. Some initiatives were in progress under the last Labour government; for instance, in 2023 in Auckland ‘equity adjustor score’ was piloted. This incorporates five additional factors not currently included in the existing ‘Clinical Priority Assessment Criteria’ for prioritizing surgical waitlists: clinical priority, waitlist duration, ethnicity, deprivation, and rurality [26]. This is the first time a sociodemographic perspective has been applied to surgical waitlists in NZ. It is clear that a one size fits all approach to surgical waitlists is not working, given the inequities we observed in our study. Differences in time to surgery based on deprivation were influenced by ethnicity in this study, the inclusion of both ethnicity and deprivation in this tool could represent a meaningful step forward. Another potential solution to address this inequity include increasing funding to recruit more cancer nurse specialists who play a key role in co-ordination of cancer treatments for patients.

### Strengths and limitations

This study provides important information on inequities by deprivation level in time to breast cancer surgery for women with early-stage breast cancer, across four urban centres in NZ. Data from *Te Rēhita Mate Ūtaetae* facilitated a comprehensive analysis; the register has a less than 1% withdrawal rate and is representative of 99% of eligible cancer cases [3, 11]. Few women (*n* = 722) were excluded due to missing deprivation status, and there was good representation across all deprivation quintiles. It contains more detail on tumour and treatment factors compared to other national databases [11]. Study limitations must be considered. Our exposure of interest, deprivation was measured using the NZDep index which is an *area*-based measure of deprivation rather than a measure of *individual* level of deprivation [12]. From 2000 to 2020 *Te Rēhita Mate Ūtaetae* only covered four large urban centres, and therefore women living rurally are underrepresented in our sample [3]. In NZ, there are a greater proportion of more deprived people living rurally, and therefore this study has not captured these women, and inequities may indeed be greater if we consider those living rurally, from more deprived areas, at greater distances from specialist breast cancer surgery centres [27]. Importantly, this study could not evaluate the historical influence of colonisation which undoubtedly is a key factor contributing to the inequities observed in this study. Additionally, the completeness of *Te Rēhita Mate Ūtaetae* for comorbidities is limited (recorded for 25%) and comorbidity may influence time to surgery, if further specialist input is required pre-operatively. However, sensitivity analysis with adjustment for CCI did not significantly change the results for either outcome.

## Conclusions

Almost half of women in NZ with breast cancer did not receive surgery within the recommended national 31-day target. Women in the most deprived group represent an underserved group experiencing a greater delay to surgery, mainly contributed to by ethnicity and treatment facility type. Implementation of the FCT guidelines in 2012, have not standardised time to surgery. Our findings underscore the need for policies addressing the timeliness and equitability of treatment for breast cancer.

## Supplementary Information

Below is the link to the electronic supplementary material.

Supplementary file1 (DOCX 24 KB)

## Data Availability

The datasets analysed during the current study are not publicly available to protect privacy and confidentiality. Data requests for de-identified data can be made online through *Te Rēhita Mate Ūtaetae* (The NZ Breast Cancer Foundation National Register).
