# Supplementary material for: Disparities in time to breast cancer surgery in New Zealand by level of neighbourhood deprivation: a population-based study
Source: Cancer Causes Control. 2025 Jul 22;36(11):1499–510. doi: 10.1007/s10552-025-02032-0 (PMC12578680; doi:10.1007/s10552-025-02032-0)
Supplement: Supplementary file 1 — Supplementary file1 (DOCX 24 KB) [file 10552_2025_2032_MOESM1_ESM.docx]

**Supplementary tables**

**Supplementary table 1. Census variables included in NZDep2018**

| **NZDep2018^a^** |
| --- |
| People with no access to Internet at home |
| People aged 18-64 receiving a means tested benefit |
| People living in equivalised households with income below an income threshold |
| People aged 18-64 who are unemployed |
| People aged 18-64 without any qualifications |
| People not living in their own home |
| People aged under 65 living in a single parent family |
| People living in equivalised households below a bedroom occupancy threshold |
| People living in dwellings that are always damp and/or have mould greater than A4 size |

1. The variables differ slightly by census year, 2018 is shown here as an example [12].

**Supplementary table 2. Categorisation of variables**

| **Domain** | **Covariates** | **Categorisation^a^** | | **Comments / Definitions** |
| --- | --- | --- | --- | --- |
|  |  | <45 years  **≥ 45 to ≤ 69 years**  >69 years | | Reference group is the screening age-group |
| Demographic | Age |  |  | Reference group is the screening age-group^b^ |
|  | Region | **Auckland**  Waikato  Christchurch  Wellington | Main urban area \| Minor urban area \| Secondary urban area \| Inlet  \| Inland Water  Rural \| Rural centre  Tis N0 M0  T1 N0 M0  T0 N1 M0 \| T1 N1 M0 \| T2 N0 M0  T2 N1 M0 \| T3 N0 M0  T0 N2 M0 \| T1 N2 M0 \| T2 N2 M0 \| T3 N1 M0 \| T3 N2 M0  T4 Any N M0  Any T N3 M0  Any T Any N M1  Low  Intermediate  High  8500/3 – Invasive carcinoma / Ductal no special type / Invasive ductal carcinoma with medullary features \| 8522/4 – Pleiomorphic ductal carcinoma \| 8211/3 – Tubular ductal carcinoma  8520/3 – Invasive lobular carcinoma \| 8035/3 – Lobular carcinoma with osteoclast giant cells |  |
|  | ^Ethnicity^ | **NZ European**  Māori  Asian  Pacific |  |  |
|  | Area of Residence | **Urban**  Rural  Unknown |  | Based on NZ Statistics Rural/Urban classification^50^  Urban includes: main urban area \| minor urban area \| secondary urban area \| inlet  Rural includes: rural \| rural centre |
|  |  |  |  |  |
| Mode of diagnosis | Mode of diagnosis | **Screened**  Symptomatic |  |  |
|  |  |  |  |  |
|  |  |  |  | American Joint Commission on Cancer 7^th^ edition – s |
| Tumour factors | TNM stage | 0  **1**  2a  2b  3a  3b  3c  4 |  | American Joint Commission on Cancer 7^th^ edition^48^ – see Table 2 for definitions |
|  | Grade | **1**  2  3  Unknown |  |  |
|  | Morphology | **Ductal**  Lobular  Mixed  Other  Unknown |  |  |
|  | ER/PR | **ER+/PR+**  ER+/PR-  ER-/PR+  ER-/PR-  Unknown, |  |  |
|  | HER | **Negative**  Equivocal  Positive  Unknown |  | Immunohistochemical analysis (IHC) used as primary assay; Fluorescence in-situ hybridization used for IHC equivocal results |
|  |  |  |  |  |
| Treatment facility type | Treatment facility type | **Public**  Private  Unknown |  |  |
|  |  |  |  |  |
| Treatment factors | Radiotherapy | **Yes**  No  Unknown |  |  |
|  | Locoregional therapy | **BCS with RT** Wide local excision \| lumpectomy  BCS without RT Wide local excision \| lumpectomy  Mastectomy | |  |
|  | Systemic therapy | **Yes**  No | Chemotherapy \| Endocrine therapy \| Biologics |  |

**Supplementary table 3. Sensitivity analysis - logistic regression models for time to surgery, by deprivation including adjustment for comorbidities**

| **Model** | **NZ Dep 1-2**  (n=1,142) | **NZ Dep 3-4**  (n=1,143) | **NZ Dep 5-6**  (n=1,090) | **NZ Dep 7-8**  (n=1,036) | **NZ Dep 9-10**  (n=707) |
| --- | --- | --- | --- | --- | --- |
|  | **Reference** | **OR (95% CI)** | | |  |
| Maximally adjusted^a^  Maximally adjusted + comorbidity score^b^ | 1.00  1.00 | 1.18 (0.99, 1.42)  1.18 (0.99, 1.42) | 1.05 (0.88, 1.27)  1.05 (0.87, 1.26) | 1.23 (1.01, 1.49)  1.22 (1.01, 1.48) | 1.38(1.10, 1.74)  1.38 (1.10, 1.73) |

1. Maximally adjusted model includes adjustment for 1) demographic factors – age (<45 years, ≥45 to ≤ 69 years (screening age in NZ), >69 years), region, area of residence (rural/urban), 2) mode of diagnosis (screened/symptomatic which includes public and private), 3) tumour biology factors – TNM stage (1 – 3a) **,** grade (low, intermediate, high, unknown), histology (ductal, lobular, mixed, other, unknown), oestrogen and progesterone receptors (ER and PR) and human epidermal growth factor receptors (HER), 4) treatment facility for surgery (public/private) and 5) treatment factors – radiotherapy and systemic therapy
2. Comorbidity score using Charlson Comorbidity Index categorized as: 0, 1-2, 3-4 and ≥5 [13].
